# Retrospective charts for reporting, analysing, and evaluating disaster emergency response: a systematic review

**DOI:** 10.1186/s12873-024-01012-y

**Published:** 2024-05-31

**Authors:** Pengwei Hu, Zhehao Li, Jing Gui, Honglei Xu, Zhongsheng Fan, Fulei Wu, Xiaorong Liu

**Affiliations:** 1 Department of Health Service, School of Public Health, Logistics University of People’s Armed Police Force, Tianjin, China; 2 Department of Health Training, Second military medical University, Shanghai, 200433 China; 3 Department of Research, Characteristic Medical Center of People Armed Police, Tianjin, China; 4 Medical Security Center, The No.983 Hospital of Joint Logistics Support Forces of Chinese PLA, Tianjin, China; 5 School of Nursing, Fudan University, Shanghai, China

**Keywords:** Retrospective charts, Emergency response, Disaster, Report

## Abstract

**Objective:**

Given the frequency of disasters worldwide, there is growing demand for efficient and effective emergency responses. One challenge is to design suitable retrospective charts to enable knowledge to be gained from disasters. This study provides comprehensive understanding of published retrospective chart review templates for designing and updating retrospective research.

**Methods:**

We conducted a systematic review and text analysis of peer-reviewed articles and grey literature on retrospective chart review templates for reporting, analysing, and evaluating emergency responses. The search was performed on PubMed, Cochrane, and Web of Science and pre-identified government and non-government organizational and professional association websites to find papers published before July 1, 2022. Items and categories were grouped and organised using visual text analysis. The study is registered in PROSPERO (374,928).

**Results:**

Four index groups, 12 guidelines, and 14 report formats (or data collection templates) from 21 peer-reviewed articles and 9 grey literature papers were eligible. Retrospective tools were generally designed based on group consensus. One guideline and one report format were designed for the entire health system, 23 studies focused on emergency systems, while the others focused on hospitals. Five papers focused specific incident types, including chemical, biological, radiological, nuclear, mass burning, and mass paediatric casualties. Ten papers stated the location where the tools were used. The text analysis included 123 categories and 1210 specific items; large heterogeneity was observed.

**Conclusion:**

Existing retrospective chart review templates for emergency response are heterogeneous, varying in type, hierarchy, and theoretical basis. The design of comprehensive, standard, and practicable retrospective charts requires an emergency response paradigm, baseline for outcomes, robust information acquisition, and among-region cooperation.

**Supplementary Information:**

The online version contains supplementary material available at 10.1186/s12873-024-01012-y.

## Introduction

The global incidence of disasters remains high. According to Centre for Research on the Epidemiology of Disasters (CRED), a total of 367 major natural disasters and more than 150 technological disasters occurred world wide in 2021, causing 10,492 and more than 5000 deaths respectively. (1–2) In this context, a growing body of evidence supports the positive impact of an efficient and effective emergency response on casualty outcomes, in both academic and operational fields of disaster medicine [3]. Although the modern era of organized disaster response of disaster can be traced back to the foundation of Red cross organization in 1863, it only became a distinct scientific discipline in the previous 60 years [4]. Disaster emergency management includes four stages: mitigation, preparedness, response, and recovery. Notably, the emergency response is recognised as having greatest immediate impact on disaster management outcomes [5]. This response requires a high level of scientific evidence to support performance improvement.

In evidence-based medicine, core concepts include population, interventions, comparison of outcomes, and hierarchy of evidence strength. However, given changing field conditions during disasters, ephemeral information, rumours, and security constraints, important questions in disaster medicine are not easily testable by evidence-based science [6]. Consequently, it is difficult to conduct controlled studies of disasters. Thus, a widely used methodology is retrospective chart review (RCR), which is a research design applicable to emergency medicine that utilizes pre-recorded data to validate research hypotheses [7–9]. Failures to create clearly articulated research questions, operationalize variables, develop and use standardized data abstraction forms are the common mistakes in RCR, making it difficult to compare outcomes of different exercises and to make evidence-based decisions in disaster management [10]. 

Given the urgent requirement for retrospective review of standard charts for data collection during disasters and for review in the aftermath, numerous evaluation indexes, report templates, and guidelines have been defined and published, such as the pre-hospital emergency response capacity index by Bayram and Zuabi, a data collection template for large-scale train accident emergency response by Leiba, et al., and the guidelines for reports on health crises and critical health events by Kulling P, et al. [11–13] These retrospective chart review templates were designed to allow researchers, educators, and managers to study different aspects of disaster management, by defining core concepts to evaluate the response, standardized work flow, and timelines from event occurrence to patients admission in emergency responses. A systematic study of templates for pre-hospital medical management of major events was published in 2013, revealing the limitations of existing templates in terms of validity and feasibility, such as unclear design methodology and lack of testing in real-life incidents [9]. Evidence is lacking regarding common aspects of retrospective charts that require attention and how reporting may be improved. Furthermore, numerous guidelines and templates from peer-reviewed articles and grey literature papers have been published since the 2013 review, such as The Health Care Coalition Surge Estimator Tool from the Administration for Strategic Preparedness and Response, after-action debriefing from Federal Emergency Management Agency, and emergency response and assessment team rapid assessment tool Association of Southeast Asian Nations [14–16]. 

This systematic review identifies existing retrospective chart review templates for reporting disaster emergency responses worldwide and provides a comprehensive assessment of these charts using content analysis. This provides a knowledge background for designing and updating widely accepted retrospective charts. The protocol is registered in PROSPERO (374,928).

## Methods

### Search strategy and criteria

To limit the scope of the review, this study focused only on the emergency response phase extending from a disaster occurrence to definitive patient treatment [5]. First, the Population, Intervention, Comparison, Outcomes, and Study Design (PICOS) model was used to shape the study question and build the search strategy. Searches were conducted using Cochrane Library, PubMed, and Web of Science to find peer-reviewed papers published before July 1, 2022, with keywords and MeSH terms related to disaster and emergency response (Supplemental Table S1 and Table S2). In addition, references from the selected articles, and prior systematic reviews were screened to identify additional relevant articles. Second, 29 pre-identified governmental, non-governmental, academic, and professional association websites and emergency-related registries stratified by World Health Organization (WHO) region were searched for published emergency response-related report forms, templates, guidelines, checklists, and data dictionaries available as of July 1, 2022 (Supplemental Table S3).

Peer-reviewed articles and grey literature were eligible if they met the following inclusion criteria: (i) the study object was an emergency response to natural, technical and social disasters, all extent of disasters from community to worldwide were included; (ii) the study designed at least one of the following types of retrospective tools: a report, a data collection template, guidelines, a checklist, a consensus, a questionnaire, or an index group with specific items for emergency response; and (iii) the study used verified specific retrospective tools to perform research related to emergency response. Papers were excluded if they met the following exclusion criteria: (i) the study only provided a theoretical frame without specific items under each concept category; (ii) any items were missing despite contacting authors to obtain the omitted information; and (iii) the study focused on an epidemiological emergency. The search, screening, and data extraction were performed independently by two reviewers (PW Hu and J Gui); any disagreements were resolved through discussion with a third investigator (FL Wu).

### Data analysis

To analyse the characteristics of the rich text objects from the included articles or grey literature, text analysis was conducted, including measures of semantics, indicators, and information acquisition, using the following steps. (i) Clear original taxonomy concepts and items under each of the concept dimensions related to health facilities’ emergency responses were extracted and included in the text analysis. (ii) For semantic measures, a theoretical frame was built to label and categorise the included items that described the time, area, action, and resource dimensions of the emergency response, consistent with the classic emergency response paradigm. Here, the ‘time’ dimension signifies the key intervals extending from the beginning of the incident to the period when the surviving victims are being treated in the hospital. The ‘area’ dimension includes four important casualty tactical emergency care zones; specifically, a hot zone, a warm zone, an en route zone, and an in-hospital zone [17]. The ‘action’ dimension includes incident command, safety and security, hazard assessment, triage and treatment (including patient tracking), and evacuation according to the mass casualty incident management framework generated by the National Disaster Life Support (NDLS) Program [18]. The ‘resource’ dimension represents the evaluations of surge capacity in the included studies; thus this dimension more specifically includes systems, spaces, staff, supplies, events, and consumption, as per ‘the science of surge’ [19, 20] (this theoretical framework is detailed in Supplemental Tables S4–S7 and Supplemental Figure S1). Four types of indicator measures were defined to categorise the items, and three information acquisition methods were identified to measure the feasibility of the included charts (these criteria are defined in Supplemental Tables S8–S9). Next, (iii) three of the current study’s authors (PW Hu, ZH Li, and J Gui) individually sorted included items using the above pre-defined taxonomy. When the three researchers could not reach consensus, a subject-matter expert was consulted. Finally, (iv) the number of items placed in each category was calculated, and text visualisation technology was used to present among-study heterogeneity (Supplemental Method).

Assessment of risk of bias (quality appraisal) was conducted using a checklist designed by the authors prior to data collection. This checklist was based on the authors’ assumptions of the data relevant to retrospective chart reports. Two of the current study’s authors (HL Xu and ZS Fan) individually assessed the risk of bias using the checklist; a subject-matter expert was consulted when consensus not reached.

## Results

**Fig. 1:**
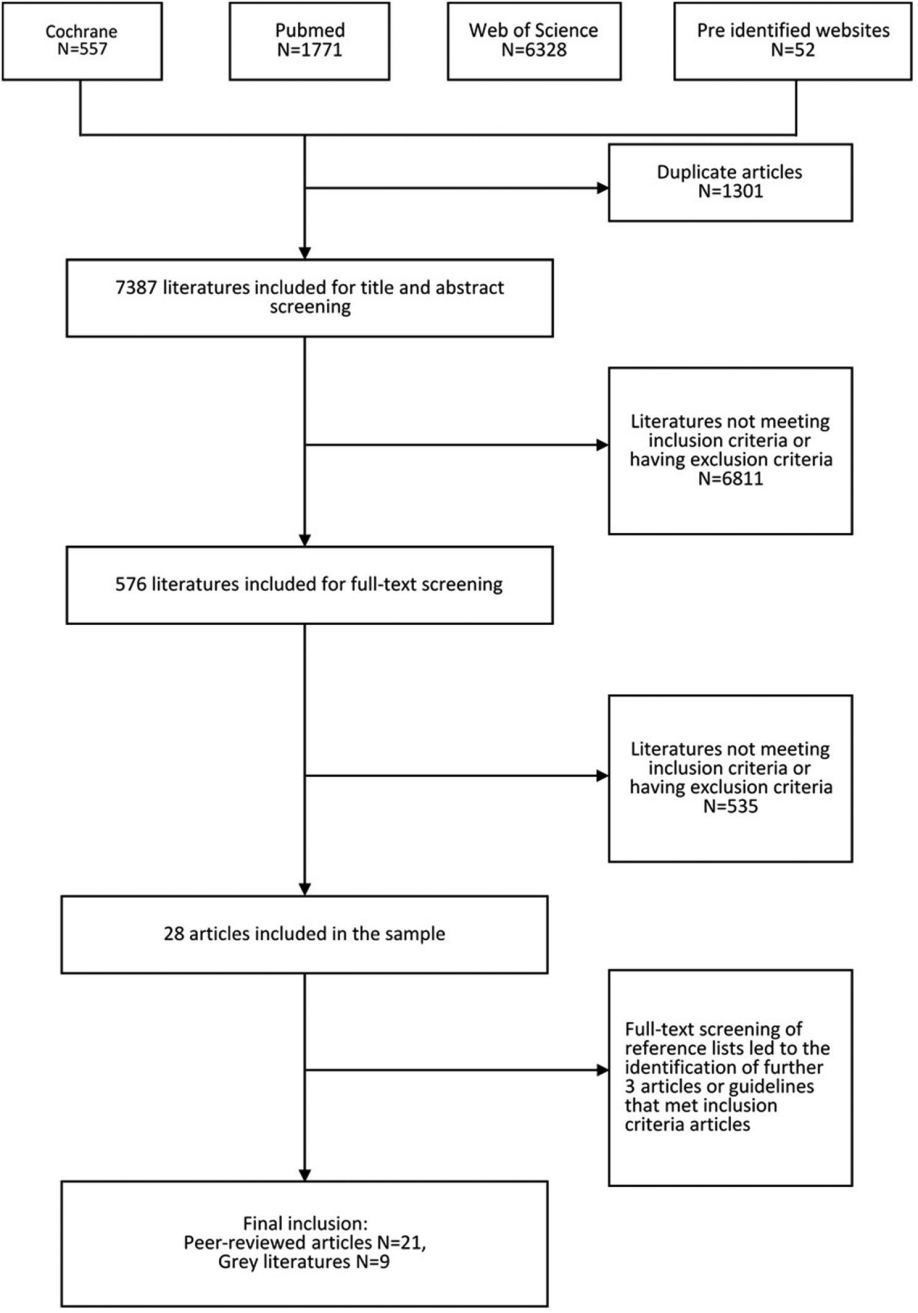
Study selection flow chart

The analysis included 4 index groups, 12 guidelines, and 14 report formats (or data collection templates) from 21 peer-reviewed articles and 9 grey literature papers [5, 6, 21–16], comprising > 2000 specific items (Fig. 1). The characteristics of the included papers are shown in Table 1. A total of 26 papers stated the methodology used to design the retrospective chart, 18 of which were based on group consensus. One set of guidelines and one report format were created for an entire health system while 23 papers focused on emergency systems and the remaining papers focused on hospitals. Eight papers mentioned the specific type of disaster, including chemical, biological, radiation, nuclear (CBRN), mass burn casualty, and mass casualty incidents involving paediatric patients. Only 10 papers revealed the country or region to which the charts were applied; specifically, 2 were used in the United States, 2 in Germany, 1 in Sweden, 1 in the Netherlands, 1 in Australia, 1 in Israel, 1 in France, 1 in southeast Asia, and 1 worldwide. Quality assessment (quality appraisal) of the papers showed that most peer-reviewed articles clearly stated the methodology and data collection procedure, while most grey literature was initiated by a department, professional, or association. All of the included papers did not indicate that there was a pilot study of the retrospective chart review templates, and only 4 templates were used in other publications (Supplementary Table S10).

**Table 1:** Characteristics of the peer-reviewed articles and grey literature included in the systematic review

Study/Publisher	Year	Agency/Agent	Country/Region	Specific type of event	Result	No. of categories	No. of items	Methodology
Peer-reviewed articles								
Thomasian, et al. [21]	2021	Hospital	United state	UM	Index group	4	24	Delphi study
lessons, T., et al. [22]	2020	Emergency system	Germany	Terrorist attack	Guideline	7	39	Experts panel consensus
Khajehaminian, et al. [42]	2020	Emergency system	UM*	UM	Guideline	5	51	Delphi study
Wurmb, et al. [23]	2018	Emergency system	Germany	Terrorist attack	Report format	13	136	Experts panel consensus
Hall, et al. [24]	2018	Emergency system	UM	Mass casualty incident	Index group	NA**	11	Delphi study
Olivieri, et al. [25]	2017	Hospital	UM	CBRN incident	Report format	6	59	Delphi study
Adini, et al. [26]	2015	Hospital	UM	UM	Guideline	NA	9	Delphi study
Fattah, et al. [27]	2014	Emergency system	UM	UM	Report format	NA	80	Experts panel consensus
Daftary, et al. [28]	2014	Health system	UM	UM	Guideline	5	37	Delphi study combined with expert panel consensus
Rådestad, et al. [29]	2013	Emergency system	Sweden	UM	Guideline	8	77	Delphi study
Debacker, et al. [5]	2012	Emergency system	UM	UM	Report format	15	90	Delphi study combined with expert panel consensus
Bayram and Zuabi [11]	2012	Emergency system	UM	UM	Index group	2	5	Concept modeling
Kulling, et al. [12]	2010	Emergency system	UM	UM	Guideline	8	21	Experts panel
Juffermans and Bierens [30]	2010	Emergency system	Netherlands	UM	Guideline	NA	20	Structural case series analysis
Bradt and Aitken [6]	2010	Emergency system	Australia	UM	Guideline	7	16	Concept modeling
Leiba, et al. [13]	2009	Emergency system	Israel	UM	Report format	3	13	Unmentioned
Lennquist [31]	2008	Emergency system	UM	UM	Guideline	18	78	Unmentioned
Belmont, et al. [32]	2004	Emergency system	United state	UM	Guideline	5	214	Unmentioned
Rüter, Anders and Vikström, Tore [33]	2003	Emergency system	French	UM	Index group	NA	11	Multi-stage concept modeling
Villarreal [34]	1997	Emergency system	UM	UM	Report format	13	126	Unmentioned
Ricci and Pretto [35]	1991	Emergency system	UM	UM	Guideline	4	42	Concept modeling
**Grey literature**								
WHO hospital emergency response checklist [36]	2011	Hospital	World-wide	UM	Guideline	9	91	Experts panel consensus
National Emergency Medical Services Information System Data Dictionary V3.5.0^35^	2021	Emergency system	United state	UM	Report format	NA	640	Unmentioned
Federal Emergency Management Agency after-action debriefing [14]	2012	Emergency system	United state	UM	Report format	NA	8	Experts panel consensus
ASPR TRACIE Health Care Coalition Surge Estimator Tool: Hospital Data Collection Form***^37^	2019	Hospital	United state	UM	Report format	NA	17	Experts panel consensus
Healthcare Coalition Radiation Emergency Surge Annex Template [38]	2019–2023	Emergency system	United state	Radiation incident	Report format	NA	26	Experts panel consensus
Healthcare Coalition Pediatric Surge Annex Template [39]	2019–2023	Emergency system	United state	Mass casualty incident involve mass pediatric injuries	Report format	NA	26	Experts panel consensus
Healthcare Coalition Chemical Emergency Surge Annex Template [40]	2019–2023	Emergency system	United state	Chemical emergency	Report format	NA	26	Experts panel consensus
Healthcare Coalition burn Surge Annex Template [41]	2019–2023	Emergency system	United state	Mass burn casualty incident	Report format	NA	26	Experts panel consensus
Association of Southeast Asian Nations Emergency Response and Assessment Team Rapid assessment tool [16]	2018	Health system	Southeast Asian	UM	Report format	NA	9	Experts panel consensus

^*^: Unmentioned; ^**^: Not Applicable; ^***^: Assistant secretary for preparedness and response.

**Fig. 2:**
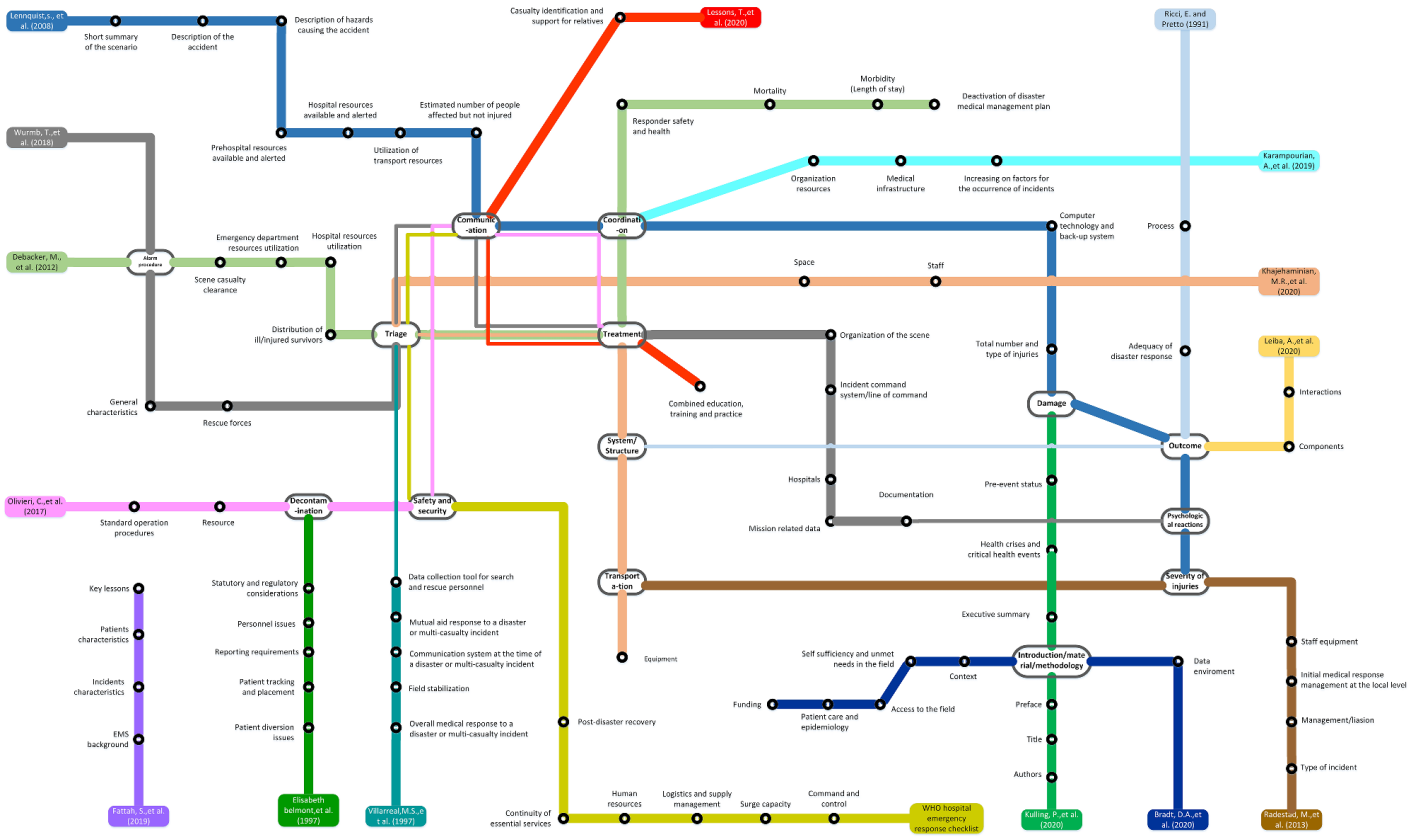
Taxonomy of the included retrospective charts

A total of 123 categories and 1210 specific items about emergency responses were included in the text analysis. The categories of the items highly varied across the papers; however, many papers commonly referred to the following 13 concepts. The most mentioned categories were ‘treatment’ and ‘communication’, which were evident in 5 studies, followed by ‘triage’ and ‘coordination’ (used by 4 studies). The text visualisation in Fig. 2 presents the categories common to papers, including ‘triage’, ‘treatment’, ‘cooperation’, and ‘communication’. The categories of the guidelines used by Lennquist et al. (2004) demonstrated the most overlap with other studies, including ‘communication’, ‘coordination’, ‘damage’, ‘outcome’, ‘psychological reactions’, and ‘severity of injuries’ [31] (Fig. 2).

**Fig. 3:**
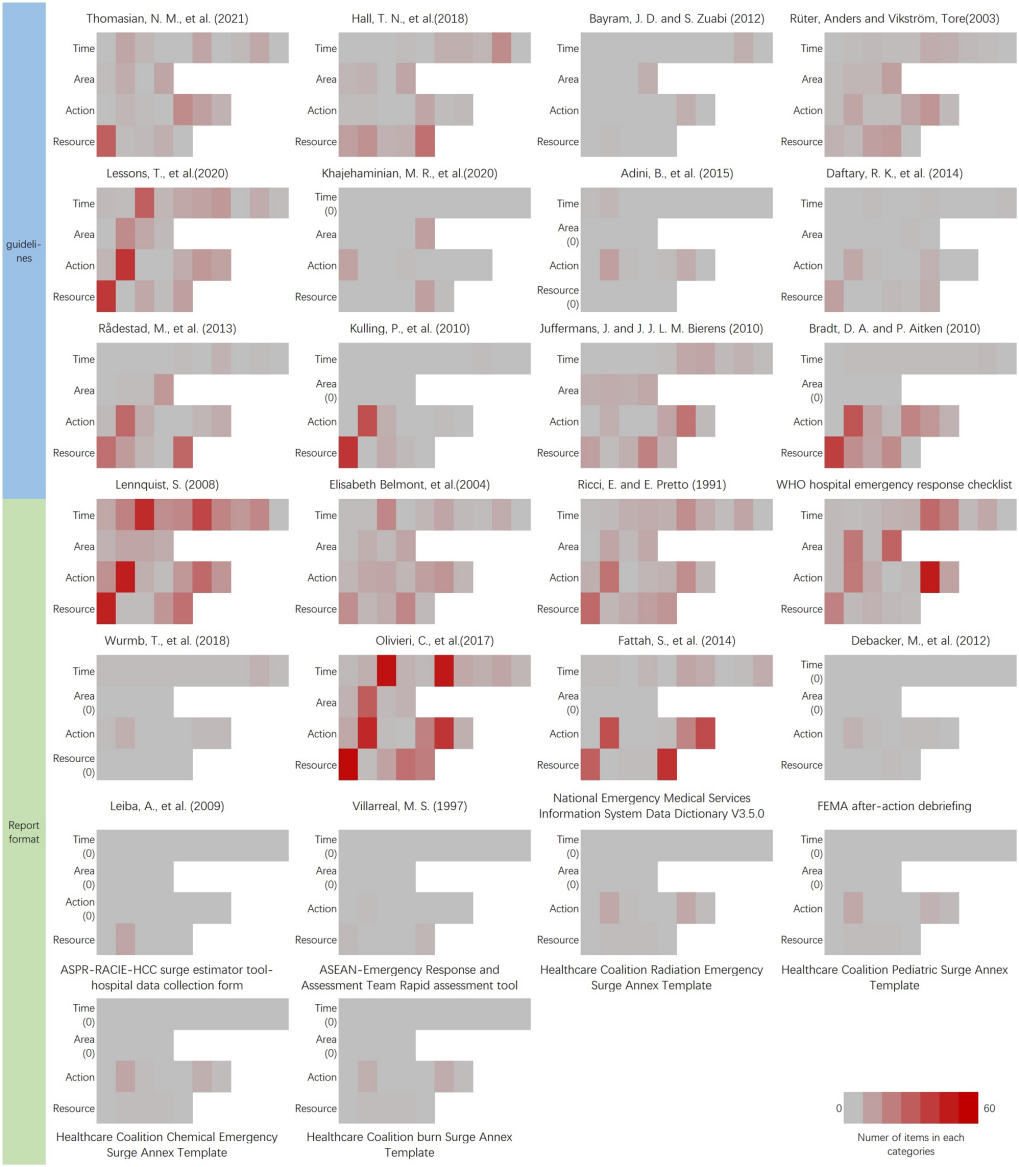
Literature fingerprint of included papers

**Fig. 4:**
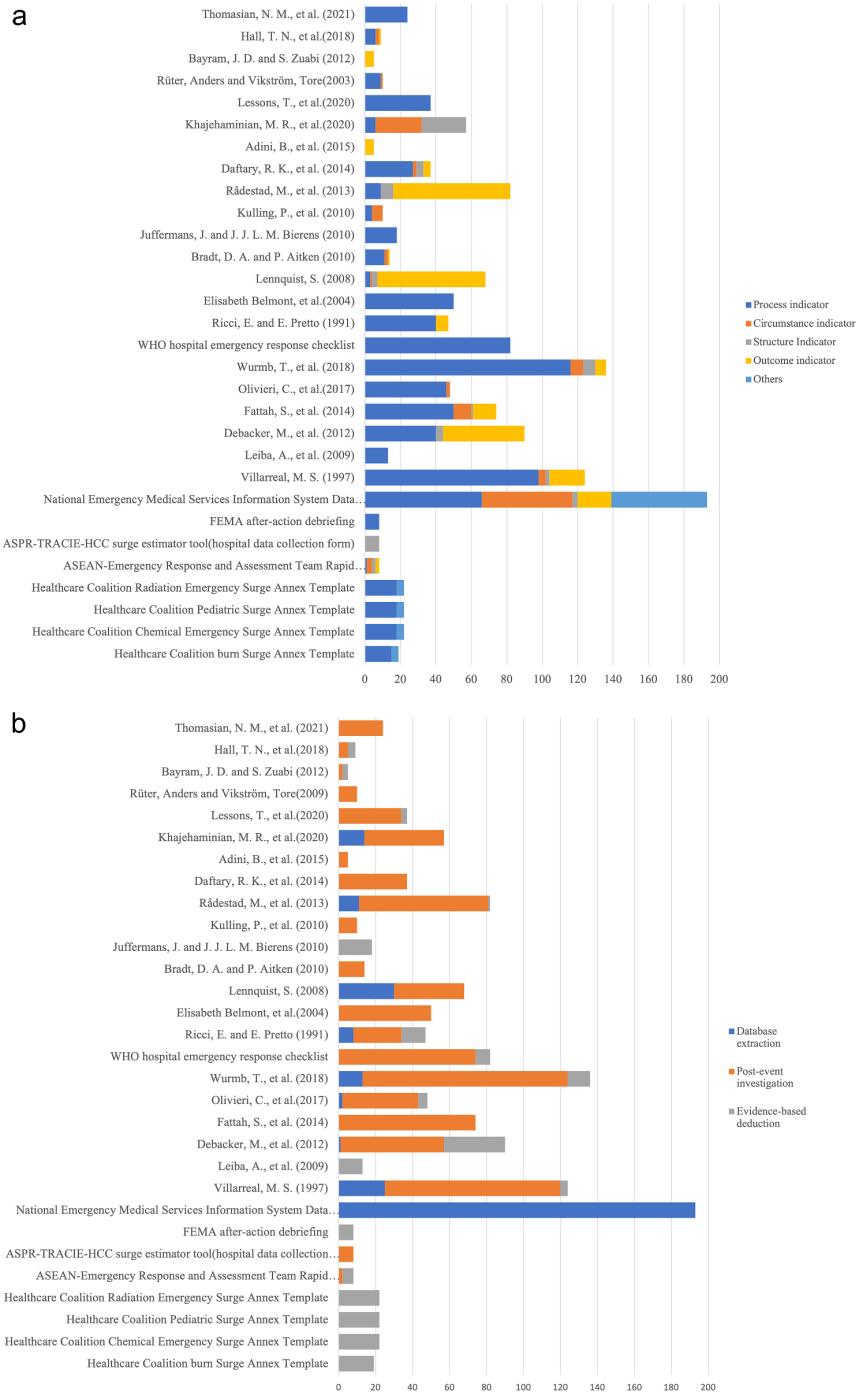
Distribution of indicator type and information acquisition methodology among the included papers, **a** shows the distribution of the indicators, **b** shows the method of information acquisition

Regarding the semantic analysis, 720 items were categorised within the time dimension, 271 within the area, 1033 within the action, and 899 within the resource. Specifically, 2 index groups, 8 guidelines, and 5 report formats were common to all four response dimensions (the time, area, action, and resource). The most frequent categories under the time dimension were on-site care and on-site command and control phases (183 and 163 items, respectively). The treatment area of most concern was the indirect threat zone (110 items), while less attention was paid to the direct threat zone (21 items). Almost all papers mentioned the ‘action’ and ‘resource’ dimensions, except one report. Regarding the ‘action’ dimension, most items were classified into ‘incident command’ (393 items), followed by ‘treatment and triage (plus tracking)’ (281 items), and ‘support’ (141 items). Regarding the ‘resource’ dimension, most items were sorted into the ‘system’ category (417 items; see Supplemental Tables S11–S14). The indicator type analysis revealed 833 expressions of process indicators, 256 outcome indicators, 117 circumstance indicators, and 66 structure indicators (Supplemental Table S15). Regarding the datatype, 884 items acquire data as text, symbol, or combination or them; 270 items collect data as number; 171 items collect data as time while 17 items acquire location (Supplemental Table S16). We also analyzed the information acquisition method, 957 items involved data collection using a post-event investigation, 299 using database extraction, and 86 using evidence-based deduction (Supplemental Table S17). Heterogeneity among studies was observed through visual inspection of bar-charts of papers, plotting text semantics, indicator types, and information acquisition methods (Figs. 3 and 4).

## Discussion

Consistent data can be collected using standard retrospective charts for emergency response that include well-defined and clearly articulated items. Such charts facilitate communication among stakeholders and beneficiaries as to whether essential standards are being met and can link policy to action [10]. To assess the current state of emergency response reporting, this study systematically reviewed 30 peer-reviewed articles and grey literature papers on emergency response report chart review templates. Most studies were based on group consensus methods, which comprehensively integrate the knowledge backgrounds of experts in relevant fields in ways that are highly relevant to the emergency response process. However, a high level of heterogeneity among these retrospective chart review templates hinders their wide application across different countries or regions. The text visualisation used in the present study suggests that the heterogeneities may arise because the included chart review templates were designed as different types, suitable for different hierarchies, and based on different theoretical paradigms. Additionally, assessment of the risk of bias in the papers indicated that high heterogeneity might also be attributed to the lack of research collaboration, unclear methods, and lack of extrapolation [43]. 

It is essential that a widely acceptable retrospective chart template is constructed based on consensus regarding the theoretical paradigm and taxonomy of items. The text visualisation of the categories of the included items revealed that each paper’s taxonomy was independent of the others’, and the theoretical paradigm used to design the chart review templates in each paper was rarely mentioned. Although some theoretical models related to emergency response were constructed by professional associations in recent years, such as ‘science of surge’ and ‘DISASRTER’, they are not widely used in the construction of retrospective charts reviews [17, 18, 44]. There exist theories that were constructed from different perspectives, such as response capability, (19–20) course of action [18], or the elements of a Utstein-style templeate [5]. A novel and comprehensive paradigm that synthesises these ideas is required to further develop and guide chart design.

We explored the commonalities and divergence among researchers when designing the retrospective charts through text semantic analysis. Regarding the definition of key intervals of the emergency response, the results revealed that researchers pay most attention to responses in the on-site care and on-site command and control phases, which immediately impact casualty care, although there is currently no widely accepted model of the chronological sequence of EMS response and care. Only 2 articles in this study had a defined response timeline, but the response timeline was not uniform between these two studies. These findings reflect the fact that most EMS systems collect time data that were empirically developed based on arbitrary concepts and ease of data collection. For the treatment area, the items designed by the researchers primarily focused on the indirect threat zone; less attention was paid to the direct threat zone, which greatly impacts the treatment of the people injured in a disaster. Accordingly, a lack of retrospective data in this area will hinder the quality improvement of pre-hospital care. This contradiction may be caused by the prioritisation of treatment in direct threat zones, which causes response information management to be relatively ignored [42]. All papers, except one report, considered the ‘action’ and ‘resource’ dimensions, indicating that researchers are primarily concerned with response action and resource use. The broad consensus that information related to ‘incident command’, ‘treatment and triage (plus tracking)’, and ‘support’ should be merged in the chart review templates, suggests that these three action classifications account for most emergency response processes and have an important impact on research. Meanwhile, numerous items were sorted within the ‘system’ dimension (based on the science of surge), which comprised the sub-components of ‘plan’, ‘command’, ‘communication’, ‘coordination’, and ‘cyber security’, which places a great amount of information in the ‘system’ dimension. Thus, it is necessary to standardise the items under ‘system’ to create widely accepted retrospective charts for emergency response.

Indicator type notably reflects the application scope and function of a retrospective chart review template. The popularity of process indicator items indicates that emergency response involves dynamic management. Due to the lack of recognised benchmark standards for evaluating emergency response, outcome indicators have the potential to serve as gold standards, which can be verified through cohort studies [45–47]. 

Retrospective data collection in emergency response can require complicated detective work, for instance, to overcome the patients remembrance deviation, infer occurrence time, and calculate the consumption. Patients are often transported to several different hospitals, making patient-specific data collection difficult [48]. Improvement of the feasibility of retrospective chart review templates could mitigate this process by improving robustness of the data acquisition method. Among the included items, interviews were the most popular way to obtain data with the advantage to easily acquire data. The feasibility of the chart review template may be improved through the comprehensive use of monitoring systems, pre-hospital emergency systems, intelligent wearable devices for situational awareness, and capturing situational awareness information by specific items [49, 50]. Further, obtaining permission from an organisation to collect data may be facilitated by referring to a specific guideline or template [51, 52]. 

Although a prior systematic study of templates for reporting prehospital medical management of major incidents was published in 2013, it had several limitations. The current study adds to the work of this 2013 study in several ways. First, it expanded the scope by conducting a systematic review of reporting for extensive emergency response, rather than just major accidents. Additionally, it conducted a detailed content analysis, integrated multiple classical theoretical backgrounds, and constructed a category framework to conduct an in-depth analysis of text-rich data to excavate the elements of emergency response to which researchers are generally attentive and how reporting may be improved.

However, the current study still had several limitations. For instance, since the included papers were only published in English, papers from non-English-speaking regions, such as Africa, China, and Russia, were not considered. Additionally, due to the difficulty of quantifying the text-rich data, and a lack of some key variables, such as the regions of application of the chart review template and the specific events of interest, subgroup analysis was not performed to explore the exact sources of heterogeneity.

## Conclusion

This study confirmed that existing retrospective chart review templates for emergency response continue to have large heterogeneity. Moving forward, data guidelines must be standardised to enable the comparison of events among countries. This would require different regions to cooperate in the design of comprehensive, standard, comparable, and feasible tools based on their own emergency response organisations.

### Electronic supplementary material

Below is the link to the electronic supplementary material.

Supplementary Material 1

Supplementary Material 2

## Data Availability

Data is provided within the manuscript and supplementary information files.
