# Supplementary material for: Retrospective charts for reporting, analysing, and evaluating disaster emergency response: a systematic review
Source: BMC Emerg Med. 2024 May 31;24:93. doi: 10.1186/s12873-024-01012-y (PMC11140892; doi:10.1186/s12873-024-01012-y)
Supplement: Supplementary file 2 — Supplementary Material 2 [file 12873_2024_1012_MOESM2_ESM.docx]

**SUPPLEMENTAL MATERIAL**

**Retrospective tools for reporting, analyzing, and evaluating emergency response: A Systematic review**

**Index**

Supplemental Methods Page 02

Supplemental Table S1. PECOS model Page 02

Supplemental Table S2. Literature search strategy Page 03

Supplementary Table S3. Grey literature source. Page 03

Supplementary Table S 4. Theoretical framework to categorize included items. Page 05

Supplementary Figure S 1. Key interval of Emergency response in chronological order. Page 05

Supplementary Table S 5. Definition of the component in “Area”. Page 05

Supplementary Table S 6. Definition of the component in “Action”. Page 05

Supplementary Table S 7. Definition of the component in “Resource”. Page 06

Supplementary Table S8. Type of indicator. Page 06

Supplementary Table S9. Information acquisition methods. Page 06

Supplementary Table S10. Assessment risk of bias (quality appraisal) of included papers. Page 07

Supplementary Table S 11. Timeline of the included studies. Page 08

Supplementary Table S 12. Casualty care zone of the included studies. Page 09

Supplementary Table S 13. Response action of the included studies. Page 11

Supplementary Table S 14. Surge capacity of the included studies. Page 12

Supplementary Table S 15. Indicator type of the included studies. Page 14

Supplementary Table S 16. Data type of the included studies. Page 15

Supplementary Table S 17. Information acquire methodology of the included studies. Page 17

Supplemental References Page 19

**Supplemental methods**

1. Data extraction

Original taxonomy and items under each of the category of included studies were extracted and included in text analysis if met following criteria: (i)the items were related to health facilities’ emergency response; (ii)the statement of item is clear with identifiable semantic primes; (iii) If the studies used subcategories, we only extract the first tier of the taxonomic pyramid.

2.lingustic perception

Grammatical and syntactic analysis were applied. Three researchers (pw, zl, gj) of present study team individually analyzed the semantic primes, grammatical structure, and the main subject of each included items. An arbitrary mark “x” was introduced if researchers can’t make a decision for analysis on specific items. If three researchers were ambiguous or can’t reach the consensus, a subject-matter expert were consorted.

3.Theoretical framework built

A theoretical framework based on key components of a dynamic event dealing with including “time”, “area”, “action”, “resource”, and “subject”（this component were analyzed in characteristics of included literatures）combined with tactical emergency casualty care, DISATER paradigm and science of surge (supplemental table S4-S7, supplemental figure S1 ). The type of indicator, type of data value, and method of information acquire were defined to analyze the comparability and feasibility of included retrospective tools.

4.Data classification and visualization

Based on the taxonomy of theoretical framework, three researchers tabled and grouped the extracted items into the categories. Cross tables were used to analyze the included characteristics of retrospective tools. Text visualization is through concept tube map and literature fingerprint. Each original categories of items in one literature were drawn as stations and collectively constitute as a line to represent the literature. When the same categories used in different literatures, the lines crossing at the stations which visualized as an exchange station. For content visual analysis, we calculate a sequence of feature values per text based on the categorized in theoretical framework and present them as a characteristic fingerprint^1^. The concept tube map and literature fingerprint were generated using Python 3.9. The feature values were calculated as how many items sorted into each categories, with SPSS 11.0.

**Supplemental Table S1. PICOS Model**

| **P**articipants: | Emergencies identified as disasters caused by natural, technical, and human-made hazards. |
| --- | --- |
| **I**ntervention: | Acute emergency response from event occurrence to patient definitive treatment. |
| **C**omparator/**C**ontrol | Not applicable. |
| **O**utcomes | Reporting Template or checklist, performance improvement indicator or guideline, evaluation tool or guideline for emergency response on mass casualty incidents. |
| **S**tudy design | Any (prior systematic reviews and meta-analysis were not included but these articles were screened for individual studies). |

**Supplemental Table S2. Literature Search Strategy**

**Supplementary Table S 2.1 Web of science**

| Emergency incident term | Disaster(TS), Mass casualty incident（TS） or Mass casualty event（TS）or major incident(TS) or major accident(TS), Terrorist attack(TS) |
| --- | --- |
|  | AND |
| Emergency response term | repon*(TS) or rescue(TS) or reaction(TS) or action(TS) |
|  | AND |
| Study term | Report (TS), case stud*(TS), case report(TS), case serie*(TS), template(TS), guideline(TS), criteria(TS), instruction(TS), check list (TS) |

**Supplementary Table S 2.2 Pubmed**

| Emergency incident term | Disaster(Mesh), Mass casualty incidents（Mesh）, Mass casualty incident（T/A）,Mass casualty event（T/A）, major incident(T/A), major accident(T/A), Terrorist attack(T/A) |
| --- | --- |
|  | AND |
| Emergency response term | repon*( T/A), rescue(T/A), reaction(T/A),action(T/A) |
|  | AND |
| Study term | Report (T/A), case study(Mesh), case stud*( T/A), case report(T/A), case serie*( T/A), template(T/A), guideline(T/A), criteria(T/A), instruction(T/A), check list (T/A) |

**Supplementary Table S 2.3 Cochrane library**

| Emergency incident term | Disaster(Mesh), Mass casualty incidents（Mesh）, Mass casualty incident（T/A）,Mass casualty event（T/A）, major incident(T/A), major accident(T/A), Terrorist attack(T/A), |
| --- | --- |
|  | AND |
| Emergency response term | repon*( T/A), rescue(T/A), reaction(T/A),action(T/A) |
|  | AND |
| Study term | Report (T/A), case study(Mesh), case stud*( T/A), case report(T/A), case serie*( T/A), template(T/A), guideline(T/A), criteria(T/A), instruction(T/A), check list (T/A) |

**Supplementary Table S3. Grey literature source**

| WHO region | Association or Agent website |
| --- | --- |
| Americas | American Burn Association |
|  | American College of Chest Physicians |
|  | American College of Emergency Physicians |
|  | American Red Cross |
|  | Assistant Secretary for Preparedness and Response |
|  | National Transportation Safety Board (NTSB) |
|  | Association of State and Territorial Health Official |
|  | Centers for Disease Control and Prevention |
|  | Federal Emergency Management Agency |
|  | National Association of County and City Health Officials |
|  | National Center for Disaster Medicine and Public Health |
|  | Pan American Health Organization |
|  | Society for Academic Emergency Medicine |
|  | Society for Critical Care Medicine |
|  | Technical Resources, Assistance Center, and Information Exchange (ASPR TRACIE) |
|  | Yale New Haven Center for Emergency Preparedness and Disaster Response |
|  | Homeland security |
| Europe | European Society for Emergency Medicine |
|  | DisasterLit |
|  | UK government |
|  | The Major Accident Reporting System (eMARS). Brussels, Belgium. European Commission |
|  | The Accident Investigation Board Norway |
|  | The Regional Centre for Emergency Medical Research and Development in western Norway |
|  | Failure and Accidents Technical Information System |
|  | Ship/Platform Collision Incident Database (UK HSE Report) |
| Western Pacific | The ASEAN(The association of southeast Asian Nations) coordination centre for Humanitarian Assistance on disaster management (AHA centre) |
|  | Asian Disaster Preparedness Center |
| Africa | Africa regional platform for disaster risk reduction |
| Eastern Mediterranean | Mediterranean Emergency Medicine Congress |

**Supplementary Table S 4. Theoretical framework to categorize included items**

| Component | Definition | Sub-component |
| --- | --- | --- |
| Time | The item refers to semantic prime including “time, date, when, now, before, after, moment” et al, related to response interval from event occurrence to patient definitive treatment. | Event notification interval, On-site command and control activation interval, On-site command and control interval, Hospital notification interval, On-site care delivery interval, On-site care interval, Evacuation interval, Admission triage interval, In-hospital treatment interval, Referral to other health facilities interval (defined in figure s1) |
| Area | The item refers to semantic prime including “be(somewhere), there, place, location, address” et al, related to tactical emergency casualty care zone. | Direct threat zone, Indirect threat zone, Evacuation En route, In-hospital care zone |
| Action | The item refers to semantic prime including “do, happen, move, touch, treat” et al, related to the specific action by responders in emergency response. | Detective, Incident Command, Safety and security, Assess hazards, Support, Triage and Treatment( include patient tracking), Evacuation and transportation |
| Resource | The item refers to semantic prime including “something, someone, skill, have, event, patient condition” et al, related to surge capacity of emergency system | System, Staff, Space, Supply, Event and Consumption |

**Supplementary Figure S 1. Key interval of Emergency response in chronological order**

The identification of the MCI

Notification of command-and-control system

Notification of hospital

Arrival of First on-scene medical team

Arrival of forward on-scene command-and-control team

Event notification interval

Command-and-control activation interval

On-site casualty care delivery interval

Hospital notification interval

Hospital notification interval

The casualties are beginning to evacuation

The casualties arrived at hospital

Referral to other health

facilities or being discharged

On-site casualty care interval

Evacuation interval

Admission triage interval

In-hospital treatment interval

The casualties are delivered to the specific department

**Supplementary Table S 5. Definition of the component in “Area”**

| Zone | Definition |
| --- | --- |
| Direct threat zone | The area where a direct and immediate threat exists. |
| Indirect threat zone | The area where a potential threat exists, but there is no direct or immediate threat. |
| Evacuation En route | The area where casualties are transport or diverted from scene to treatment facilities. |
| In-hospital treatment zone | The area where patients are treated stably and far from threat area, including established local, regional, state, or national hospitals, trauma centres, or temporary health facilities. |

**Supplementary Table S 6. Definition of the component in “Action”**

| Components | Definition |
| --- | --- |
| Detective | Mass casualty incident identification, immediately declare to dispatch. |
| Incident Command | Establish or interface with the Incident Command System |
| Safety and Security | Immediate action steps to immediately protect responders, casualties, and public |
| Assess Hazards | Actively assess (initially and ongoing) for hazards that can harm responders, casualties, and public |
| Support | Request resources needed to effectively manage incident |
| Triage and Treatment | Initiate Triage and provide treatment to casualties with patient tracking |
| Evacuation | Transport of casualties to appropriate hospitals (avoiding overloading individual hospitals) or alternate treatment centers |

**Supplementary Table S 7. Definition of the component in “Resource”**

| Components | Definition |
| --- | --- |
| System | Planning, community infrastructure, public health, incident command, regional cooperation, communication and information flow, supply chain distribution, EMS/first responders, continuity of operations, cybersecurit |
| Space | Facilities, including medical care, storage, laboratory, mortuary, housing of staff, quality, including size, capability, location |
| Staff | Numbers, capability/skill set, expertise, stamina, psych |
| Supply | Biologics, respirators, personal protective equipment, standard supplies, food and water |
| Event and consumption | Incident type, scale, and duration; Casualty Consumption and degradation |

**Supplementary Table S8. Type of indicator**

| Types | Definition |
| --- | --- |
| Process indicator | Defined as the indicator describing activities or processes involved in emergency response management at, and is usually associated with patient outcome. |
| Circumstance indicator | Defined as the indicator describing the factors related to the event and casualties, for example type of event, the weather, road traffic, roles of responder, level of alarm, and cause of injury. |
| Structure indicator | Defined as the quantitative measure reflecting availability of resources, for example number of ambulances, involved in medical response management. |
| Outcome indicator | Defined as the result of the activities or actions, indicating whether the planned actions happened, for example reduction in morbidity and mortality of the disaster survivors , percentage of over-triage. |

**Supplementary Table S 9. Information acquisition methods**

| Methods | Definition |
| --- | --- |
| Information system database extraction, or Documentation review | The information of the items can be extracted from present information system, for example emergency information system, hospital information system or trauma registry. |
| Investigation | The information of the items can be acquired through litigant’s interviewing or investigation with structural or semi-structural feedback forms, survey, or interviews |
| Evidence-based deduction | The information of the items cannot be directly acquired through present information system, interviewing, or investigation, but can be deducted based on acquired information. |

**Supplementary Table S 10. Assessment risk of bias (quality appraisal) of included papers**

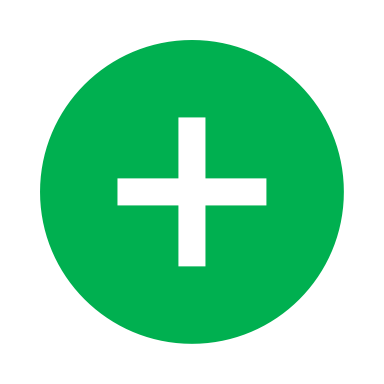
**:Yes;**
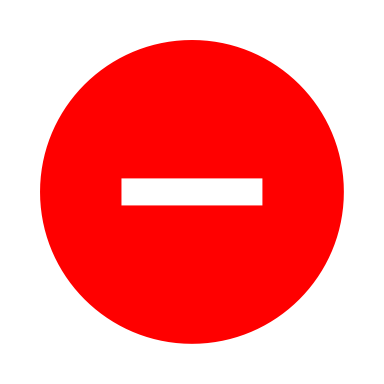
**:No;**
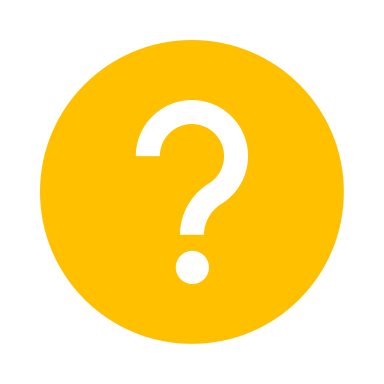
**:unclear**

**Supplementary Table S 11. Distribution of items categorized into each pre-defined time interval**

| Study | Event notification  interval | Command & control  Activation interval | On-site command &  Control interval | Hospital notification interval | On-site casualty care  Delivery interval | On-site care  intetval | Evacuation  interval | Administration  Triage interval | In-hospital treatment  interval | Referral to other  Hospital interval | NA |
| --- | --- | --- | --- | --- | --- | --- | --- | --- | --- | --- | --- |
| Thomasian, N. M., et al. (2021) | 0 | 0 | 0 | 0 | 0 | 0 | 0 | 0 | 0 | 0 | 24 |
| Lessons, T., et al.(2020) | 0 | 8 | 3 | 0 | 0 | 8 | 0 | 2 | 7 | 0 | 9 |
| Khajehaminian, M. R., et al.(2020) | 0 | 0 | 0 | 1 | 0 | 5 | 5 | 3 | 16 | 0 | 27 |
| Wurmb, T., et al. (2018) | 7 | 19 | 45 | 15 | 17 | 37 | 18 | 12 | 17 | 0 | 34 |
| Hall, T. N., et al.(2018) | 0 | 0 | 0 | 0 | 1 | 0 | 1 | 0 | 1 | 0 | 6 |
| Olivieri, C., et al.(2017) | 1 | 1 | 17 | 0 | 3 | 8 | 2 | 1 | 6 | 0 | 11 |
| Adini, B., et al. (2015) | 0 | 0 | 0 | 0 | 0 | 0 | 0 | 0 | 5 | 0 | 0 |
| Fattah, S., et al. (2014) | 1 | 2 | 5 | 6 | 5 | 15 | 6 | 1 | 8 | 0 | 42 |
| Daftary, R. K., et al. (2014) | 0 | 1 | 2 | 1 | 1 | 6 | 5 | 2 | 1 | 0 | 26 |
| Rådestad, M., et al. (2013) | 2 | 1 | 29 | 3 | 7 | 9 | 12 | 0 | 6 | 1 | 11 |
| Debacker, M., et al. (2012) | 2 | 1 | 2 | 4 | 5 | 26 | 18 | 2 | 7 | 0 | 31 |
| Bayram, J. D. and S. Zuabi (2012) | 1 | 1 | 1 | 1 | 0 | 0 | 1 | 0 | 0 | 0 | 3 |
| Kulling, P., et al. (2010) | 0 | 0 | 0 | 0 | 0 | 0 | 0 | 0 | 0 | 0 | 10 |
| Bradt, D. A. and P. Aitken (2010) | 0 | 0 | 1 | 0 | 0 | 1 | 0 | 0 | 0 | 1 | 11 |
| Rüter, Anders and Vikström, Tore(2009) | 1 | 2 | 3 | 0 | 0 | 0 | 0 | 0 | 0 | 3 | 1 |
| Lennquist, S. (2008) | 0 | 0 | 0 | 0 | 1 | 0 | 4 | 0 | 1 | 0 | 62 |
| Elisabeth Belmont, et al.(2004) | 0 | 0 | 0 | 0 | 0 | 0 | 0 | 1 | 0 | 0 | 49 |
| Villarreal, M. S. (1997) | 2 | 5 | 53 | 4 | 2 | 51 | 7 | 5 | 8 | 3 | 7 |
| Ricci, E. and E. Pretto (1991) | 0 | 0 | 0 | 0 | 1 | 7 | 8 | 2 | 4 | 1 | 28 |
| Juffermans, J. and J. J. L. M. Bierens (2010) | 1 | 3 | 0 | 0 | 0 | 0 | 0 | 0 | 0 | 0 | 14 |
| Leiba, A., et al. (2009) | 1 | 1 | 1 | 1 | 1 | 1 | 1 | 1 | 4 | 1 | 9 |
| WHO hospital emergency response checklist | 0 | 1 | 1 | 1 | 1 | 1 | 1 | 1 | 2 | 0 | 80 |
| aspr-tracie-hcc-surge-estimator-tool-hospital-data-collection-form | 0 | 0 | 0 | 0 | 0 | 0 | 0 | 0 | 0 | 0 | 8 |
| National Emergency Medical Services Information System Data Dictionary V3.5.0 | 1 | 1 | 0 | 3 | 0 | 8 | 6 | 0 | 1 | 6 | 175 |
| FEMA after-action debriefing | 0 | 0 | 0 | 0 | 0 | 0 | 0 | 0 | 0 | 0 | 8 |
| Healthcare Coalition Radiation Emergency Surge Annex Template | 0 | 0 | 0 | 0 | 0 | 0 | 0 | 0 | 0 | 0 | 22 |
| Healthcare Coalition Pediatric Surge Annex Template | 0 | 0 | 0 | 0 | 0 | 0 | 0 | 0 | 0 | 0 | 22 |
| Healthcare Coalition Chemical Emergency Surge Annex Template | 0 | 0 | 0 | 0 | 0 | 0 | 0 | 0 | 0 | 0 | 22 |
| Healthcare Coalition burn Surge Annex Template | 0 | 0 | 0 | 0 | 0 | 0 | 0 | 0 | 0 | 0 | 19 |
| ASEAN-Emergency Response and Assessment Team Rapid assessment tool-report format | 0 | 0 | 0 | 0 | 0 | 0 | 0 | 0 | 0 | 0 | 8 |
| Total | 20 | 47 | 163 | 40 | 45 | 183 | 95 | 33 | 94 | 16 | 779 |

**Supplementary Table S 12. Distribution of items categorized into each pre-defined casualty care zone**

| Study | Direct threat zone | Indirect threat zone | Evacuation En route | In-hospital treatment zone | NA |
| --- | --- | --- | --- | --- | --- |
| Thomasian, N. M., et al. (2021) | 0 | 0 | 0 | 0 | 24 |
| Lessons, T., et al.(2020) | 1 | 6 | 0 | 8 | 22 |
| Khajehaminian, M. R., et al.(2020) | 3 | 4 | 0 | 8 | 45 |
| Wurmb, T., et al. (2018) | 4 | 8 | 8 | 6 | 118 |
| Hall, T. N., et al.(2018) | 0 | 0 | 0 | 0 | 9 |
| Olivieri, C., et al.(2017) | 1 | 5 | 2 | 8 | 35 |
| Adini, B., et al. (2015) | 0 | 0 | 0 | 5 | 0 |
| Fattah, S., et al. (2014) | 0 | 8 | 2 | 7 | 57 |
| Daftary, R. K., et al. (2014) | 1 | 3 | 3 | 10 | 25 |
| Rådestad, M., et al. (2013) | 0 | 16 | 7 | 2 | 57 |
| Debacker, M., et al. (2012) | 2 | 22 | 2 | 28 | 37 |
| Bayram, J. D. and S. Zuabi (2012) | 0 | 0 | 0 | 0 | 5 |
| Kulling, P., et al. (2010) | 0 | 0 | 0 | 0 | 10 |
| Bradt, D. A. and P. Aitken (2010) | 0 | 0 | 0 | 0 | 14 |
| Rüter, Anders and Vikström, Tore(2009) | 0 | 1 | 0 | 3 | 6 |
| Lennquist, S. (2008) | 0 | 1 | 1 | 12 | 54 |
| Elisabeth Belmont, et al.(2004) | 0 | 0 | 0 | 0 | 50 |
| Villarreal, M. S. (1997) | 4 | 30 | 2 | 4 | 84 |
| Ricci, E. and E. Pretto (1991) | 5 | 6 | 5 | 7 | 32 |
| WHO hospital emergency response checklist | 0 | 0 | 0 | 0 | 82 |
| aspr-tracie-hcc-surge-estimator-tool-hospital-data-collection-form | 0 | 0 | 0 | 0 | 8 |
| National Emergency Medical Services Information System Data Dictionary V3.5.0 | 0 | 0 | 0 | 0 | 193 |
| Juffermans, J. and J. J. L. M. Bierens (2010) | 0 | 0 | 0 | 0 | 18 |
| FEMA after-action debriefing | 0 | 0 | 0 | 0 | 8 |
| Healthcare Coalition Radiation Emergency Surge Annex Template | 0 | 0 | 0 | 0 | 22 |
| Healthcare Coalition Pediatric Surge Annex Template | 0 | 0 | 0 | 0 | 22 |
| Healthcare Coalition Chemical Emergency Surge Annex Template | 0 | 0 | 0 | 0 | 22 |
| Healthcare Coalition burn Surge Annex Template | 0 | 0 | 0 | 0 | 19 |
| ASEAN-Emergency Response and Assessment Team Rapid assessment tool-repert format | 0 | 0 | 0 | 0 | 8 |
| Leiba, A., et al. (2009) | 0 | 0 | 0 | 0 | 13 |
| Total | 21 | 110 | 32 | 108 | 1099 |

**Supplementary Table S 13. Distribution of items categorized into each pre-defined response action**

| Study | Detective | Incident command | Safety and security | Access Hazards | Support | Triage, treatment  and tracking | Evacuation | NA |
| --- | --- | --- | --- | --- | --- | --- | --- | --- |
| Thomasian, N. M., et al. (2021) | 0 | 0 | 0 | 0 | 24 | 0 | 0 | 0 |
| Lessons, T., et al.(2020) | 0 | 3 | 1 | 0 | 16 | 10 | 4 | 3 |
| Khajehaminian, M. R., et al.(2020) | 1 | 1 | 0 | 0 | 8 | 1 | 2 | 44 |
| Wurmb, T., et al. (2018) | 14 | 49 | 5 | 1 | 12 | 26 | 13 | 16 |
| Hall, T. N., et al.(2018) | 1 | 2 | 0 | 0 | 2 | 0 | 1 | 3 |
| Olivieri, C., et al.(2017) | 3 | 7 | 8 | 3 | 10 | 13 | 3 | 1 |
| Adini, B., et al. (2015) | 0 | 0 | 0 | 0 | 0 | 5 | 0 | 0 |
| Fattah, S., et al. (2014) | 5 | 23 | 0 | 3 | 4 | 15 | 3 | 21 |
| Daftary, R. K., et al. (2014) | 1 | 8 | 1 | 1 | 8 | 13 | 2 | 3 |
| Rådestad, M., et al. (2013) | 3 | 42 | 0 | 0 | 6 | 12 | 9 | 10 |
| Debacker, M., et al. (2012) | 2 | 21 | 6 | 0 | 2 | 50 | 9 | 0 |
| Bayram, J. D. and S. Zuabi (2012) | 0 | 1 | 0 | 0 | 0 | 2 | 2 | 0 |
| Kulling, P., et al. (2010) | 9 | 0 | 0 | 1 | 0 | 0 | 0 | 0 |
| Bradt, D. A. and P. Aitken (2010) | 0 | 7 | 1 | 0 | 0 | 4 | 0 | 2 |
| Rüter, Anders and Vikström, Tore(2009) | 1 | 5 | 0 | 0 | 1 | 0 | 3 | 0 |
| Lennquist, S. (2008) | 2 | 26 | 6 | 0 | 0 | 3 | 6 | 25 |
| Elisabeth Belmont, et al.(2004) | 0 | 34 | 5 | 0 | 0 | 1 | 0 | 10 |
| Villarreal, M. S. (1997) | 7 | 46 | 1 | 0 | 19 | 42 | 5 | 4 |
| Ricci, E. and E. Pretto (1991) | 0 | 5 | 0 | 0 | 8 | 23 | 2 | 9 |
| WHO hospital emergency response checklist | 0 | 32 | 9 | 2 | 18 | 12 | 4 | 5 |
| aspr-tracie-hcc-surge-estimator-tool-hospital-data-collection-form | 0 | 0 | 0 | 0 | 0 | 0 | 0 | 8 |
| National Emergency Medical Services Information System Data Dictionary V3.5.0 | 0 | 34 | 0 | 0 | 1 | 18 | 35 | 105 |
| Juffermans, J. and J. J. L. M. Bierens (2010) | 0 | 10 | 1 | 0 | 1 | 6 | 0 | 0 |
| FEMA after-action debriefing | 0 | 4 | 1 | 0 | 1 | 1 | 0 | 1 |
| Healthcare Coalition Radiation Emergency Surge Annex Template | 0 | 7 | 1 | 0 | 0 | 7 | 1 | 6 |
| Healthcare Coalition Pediatric Surge Annex Template | 0 | 7 | 0 | 0 | 0 | 4 | 2 | 9 |
| Healthcare Coalition Chemical Emergency Surge Annex Template | 0 | 8 | 1 | 0 | 0 | 5 | 1 | 7 |
| Healthcare Coalition burn Surge Annex Template | 0 | 5 | 0 | 0 | 0 | 6 | 1 | 7 |
| ASEAN-Emergency Response and Assessment Team Rapid assessment tool-repert format | 0 | 1 | 0 | 0 | 0 | 0 | 0 | 7 |
| Leiba, A., et al. (2009) | 1 | 5 | 0 | 0 | 0 | 2 | 2 | 3 |
| Total | 50 | 393 | 47 | 11 | 141 | 281 | 110 | 309 |

**Supplementary Table S 14. Distribution of items categorized into each pre-defined components of surge capacity**

| Study | System | Space | Supply | Staff | Event and Consumption | NA |
| --- | --- | --- | --- | --- | --- | --- |
| Thomasian, N. M., et al. (2021) | 7 | 10 | 2 | 5 | 0 | 0 |
| Lessons, T., et al.(2020) | 29 | 1 | 2 | 5 | 0 | 0 |
| Khajehaminian, M. R., et al.(2020) | 10 | 14 | 4 | 5 | 24 | 0 |
| Wurmb, T., et al. (2018) | 48 | 1 | 1 | 13 | 24 | 49 |
| Hall, T. N., et al.(2018) | 2 | 0 | 1 | 2 | 2 | 2 |
| Olivieri, C., et al.(2017) | 15 | 3 | 6 | 17 | 2 | 5 |
| Adini, B., et al. (2015) | 0 | 1 | 0 | 0 | 0 | 4 |
| Fattah, S., et al. (2014) | 26 | 3 | 6 | 10 | 13 | 16 |
| Daftary, R. K., et al. (2014) | 4 | 2 | 10 | 11 | 0 | 10 |
| Rådestad, M., et al. (2013) | 41 | 0 | 7 | 1 | 10 | 23 |
| Debacker, M., et al. (2012) | 19 | 2 | 4 | 2 | 1 | 62 |
| Bayram, J. D. and S. Zuabi (2012) | 3 | 0 | 0 | 0 | 0 | 2 |
| Kulling, P., et al. (2010) | 0 | 0 | 0 | 0 | 10 | 0 |
| Bradt, D. A. and P. Aitken (2010) | 10 | 0 | 0 | 2 | 1 | 1 |
| Rüter, Anders and Vikström, Tore(2009) | 2 | 0 | 1 | 0 | 1 | 6 |
| Lennquist, S. (2008) | 24 | 11 | 3 | 0 | 27 | 3 |
| Elisabeth Belmont, et al.(2004) | 42 | 0 | 6 | 2 | 0 | 0 |
| Villarreal, M. S. (1997) | 55 | 1 | 9 | 24 | 20 | 15 |
| Ricci, E. and E. Pretto (1991) | 10 | 1 | 5 | 19 | 4 | 8 |
| WHO hospital emergency response checklist | 39 | 15 | 8 | 16 | 0 | 4 |
| aspr-tracie-hcc-surge-estimator-tool-hospital-data-collection-form | 0 | 8 | 0 | 0 | 0 | 0 |
| National Emergency Medical Services Information System Data Dictionary V3.5.0 | 29 | 0 | 1 | 1 | 43 | 119 |
| Juffermans, J. and J. J. L. M. Bierens (2010) | 0 | 0 | 0 | 0 | 0 | 18 |
| FEMA after-action debriefing | 0 | 0 | 0 | 1 | 0 | 7 |
| Healthcare Coalition Radiation Emergency Surge Annex Template | 0 | 1 | 1 | 1 | 0 | 19 |
| Healthcare Coalition Pediatric Surge Annex Template | 0 | 1 | 1 | 1 | 0 | 19 |
| Healthcare Coalition Chemical Emergency Surge Annex Template | 0 | 1 | 1 | 1 | 0 | 19 |
| Healthcare Coalition burn Surge Annex Template | 0 | 1 | 1 | 1 | 0 | 16 |
| ASEAN-Emergency Response and Assessment Team Rapid assessment tool-repert format | 2 | 0 | 0 | 0 | 3 | 3 |
| Leiba, A., et al. (2009) | 0 | 0 | 0 | 0 | 0 | 13 |
| Total | 417 | 77 | 80 | 140 | 185 | 443 |

**Supplementary Table S 15. Distribution of items categorized into each type of indicator**

| Study | Process indicator | Circumstance indicator | Structure indicator | Outcome indicator | Others |
| --- | --- | --- | --- | --- | --- |
| Thomasian, N. M., et al. (2021) | 24 | 0 | 0 | 0 | 0 |
| Lessons, T., et al.(2020) | 37 | 0 | 0 | 0 | 0 |
| Khajehaminian, M. R., et al.(2020) | 6 | 26 | 25 | 0 | 0 |
| Wurmb, T., et al. (2018) | 116 | 7 | 7 | 6 | 0 |
| Hall, T. N., et al.(2018) | 6 | 2 | 0 | 1 | 0 |
| Olivieri, C., et al.(2017) | 46 | 2 | 0 | 0 | 0 |
| Adini, B., et al. (2015) | 0 | 0 | 0 | 5 | 0 |
| Fattah, S., et al. (2014) | 50 | 10 | 1 | 13 | 0 |
| Daftary, R. K., et al. (2014) | 27 | 2 | 4 | 4 | 0 |
| Rådestad, M., et al. (2013) | 9 | 0 | 7 | 66 | 0 |
| Debacker, M., et al. (2012) | 40 | 0 | 4 | 46 | 0 |
| Bayram, J. D. and S. Zuabi (2012) | 0 | 0 | 0 | 5 | 0 |
| Kulling, P., et al. (2010) | 4 | 6 | 0 | 0 | 0 |
| Bradt, D. A. and P. Aitken (2010) | 11 | 2 | 0 | 1 | 0 |
| Rüter, Anders and Vikström, Tore(2009) | 9 | 1 | 0 | 0 | 0 |
| Lennquist, S. (2008) | 3 | 1 | 3 | 61 | 0 |
| Elisabeth Belmont, et al.(2004) | 50 | 0 | 0 | 0 | 0 |
| Villarreal, M. S. (1997) | 98 | 4 | 2 | 20 | 0 |
| Ricci, E. and E. Pretto (1991) | 40 | 0 | 0 | 7 | 0 |
| WHO hospital emergency response checklist | 82 | 0 | 0 | 0 | 0 |
| aspr-tracie-hcc-surge-estimator-tool-hospital-data-collection-form | 0 | 0 | 8 | 0 | 0 |
| National Emergency Medical Services Information System Data Dictionary V3.5.0 | 66 | 51 | 3 | 19 | 54 |
| Juffermans, J. and J. J. L. M. Bierens (2010) | 18 | 0 | 0 | 0 | 0 |
| FEMA after-action debriefing | 8 | 0 | 0 | 0 | 0 |
| Healthcare Coalition Radiation Emergency Surge Annex Template | 18 | 0 | 0 | 0 | 4 |
| Healthcare Coalition Pediatric Surge Annex Template | 18 | 0 | 0 | 0 | 4 |
| Healthcare Coalition Chemical Emergency Surge Annex Template | 18 | 0 | 0 | 0 | 4 |
| Healthcare Coalition burn Surge Annex Template | 15 | 0 | 0 | 0 | 4 |
| ASEAN-Emergency Response and Assessment Team Rapid assessment tool-repert format | 1 | 3 | 2 | 2 | 0 |
| Leiba, A., et al. (2009) | 13 | 0 | 0 | 0 | 0 |
| Total | 833 | 117 | 66 | 256 | 70 |

**Supplementary Table S 16. Data type of the included studies**

| Study | String^*^ | Number | Location | Time |
| --- | --- | --- | --- | --- |
| Thomasian, N. M., et al. (2021) | 24 | 0 | 0 | 0 |
| Lessons, T., et al.(2020) | 37 | 0 | 0 | 0 |
| Khajehaminian, M. R., et al.(2020) | 12 | 40 | 4 | 1 |
| Wurmb, T., et al. (2018) | 71 | 20 | 0 | 45 |
| Hall, T. N., et al.(2018) | 8 | 0 | 0 | 1 |
| Olivieri, C., et al.(2017) | 40 | 0 | 4 | 4 |
| Adini, B., et al. (2015) | 0 | 5 | 0 | 0 |
| Fattah, S., et al. (2014) | 49 | 19 | 1 | 5 |
| Daftary, R. K., et al. (2014) | 22 | 14 | 0 | 1 |
| Rådestad, M., et al. (2013) | 21 | 18 | 0 | 43 |
| Debacker, M., et al. (2012) | 14 | 53 | 0 | 23 |
| Bayram, J. D. and S. Zuabi (2012) | 0 | 5 | 0 | 0 |
| Kulling, P., et al. (2010) | 7 | 0 | 1 | 2 |
| Bradt, D. A. and P. Aitken (2010) | 12 | 0 | 0 | 2 |
| Rüter, Anders and Vikström, Tore(2009) | 10 | 0 | 0 | 0 |
| Lennquist, S. (2008) | 27 | 37 | 0 | 4 |
| Elisabeth Belmont, et al.(2004) | 50 | 0 | 0 | 0 |
| Villarreal, M. S. (1997) | 97 | 12 | 0 | 15 |
| Ricci, E. and E. Pretto (1991) | 46 | 1 | 0 | 0 |
| WHO hospital emergency response checklist | 81 | 1 | 0 | 0 |
| aspr-tracie-hcc-surge-estimator-tool-hospital-data-collection-form | 1 | 7 | 0 | 0 |
| National Emergency Medical Services Information System Data Dictionary V3.5.0 | 126 | 36 | 7 | 24 |
| Juffermans, J. and J. J. L. M. Bierens (2010) | 18 | 0 | 0 | 0 |
| FEMA after-action debriefing | 8 | 0 | 0 | 0 |
| Healthcare Coalition Radiation Emergency Surge Annex Template | 22 | 0 | 0 | 0 |
| Healthcare Coalition Pediatric Surge Annex Template | 22 | 0 | 0 | 0 |
| Healthcare Coalition Chemical Emergency Surge Annex Template | 22 | 0 | 0 | 0 |
| Healthcare Coalition burn Surge Annex Template | 19 | 0 | 0 | 0 |
| ASEAN-Emergency Response and Assessment Team Rapid assessment tool-repert format | 8 | 0 | 0 | 0 |
| Leiba, A., et al. (2009) | 10 | 2 | 0 | 1 |
| Total | 884 | 270 | 17 | 171 |

*: The string data indicates text, symbol, or combination of them.

**Supplementary Table S 17. Distribution of items categorized into each pre-defined information acquire methodology**

| Study | Database extraction | Investigation | Evidence-based deduction |
| --- | --- | --- | --- |
| Thomasian, N. M., et al. (2021) | 0 | 24 | 0 |
| Lessons, T., et al.(2020) | 0 | 34 | 3 |
| Khajehaminian, M. R., et al.(2020) | 14 | 43 | 0 |
| Wurmb, T., et al. (2018) | 13 | 111 | 12 |
| Hall, T. N., et al.(2018) | 0 | 5 | 4 |
| Olivieri, C., et al.(2017) | 2 | 41 | 5 |
| Adini, B., et al. (2015) | 0 | 5 | 0 |
| Fattah, S., et al. (2014) | 0 | 74 | 0 |
| Daftary, R. K., et al. (2014) | 0 | 37 | 0 |
| Rådestad, M., et al. (2013) | 11 | 70 | 1 |
| Debacker, M., et al. (2012) | 1 | 56 | 33 |
| Bayram, J. D. and S. Zuabi (2012) | 0 | 2 | 3 |
| Kulling, P., et al. (2010) | 0 | 10 | 0 |
| Bradt, D. A. and P. Aitken (2010) | 0 | 14 | 0 |
| Rüter, Anders and Vikström, Tore(2009) | 0 | 10 | 0 |
| Lennquist, S. (2008) | 30 | 38 | 0 |
| Elisabeth Belmont, et al.(2004) | 0 | 50 | 0 |
| Villarreal, M. S. (1997) | 25 | 95 | 4 |
| Ricci, E. and E. Pretto (1991) | 8 | 26 | 13 |
| WHO hospital emergency response checklist | 0 | 74 | 8 |
| aspr-tracie-hcc-surge-estimator-tool-hospital-data-collection-form | 0 | 8 | 0 |
| National Emergency Medical Services Information System Data Dictionary V3.5.0 | 193 | 0 | 0 |
| Juffermans, J. and J. J. L. M. Bierens (2010) | 0 | 18 | 0 |
| FEMA after-action debriefing | 0 | 8 | 0 |
| Healthcare Coalition Radiation Emergency Surge Annex Template | 0 | 22 | 0 |
| Healthcare Coalition Pediatric Surge Annex Template | 0 | 22 | 0 |
| Healthcare Coalition Chemical Emergency Surge Annex Template | 0 | 22 | 0 |
| Healthcare Coalition burn Surge Annex Template | 0 | 19 | 0 |
| ASEAN-Emergency Response and Assessment Team Rapid assessment tool-repert format | 2 | 6 | 0 |
| Leiba, A., et al. (2009) | 0 | 13 | 0 |
| Total | 299 | 957 | 86 |

Supplemental References

1. D. A. Keim and D. Oelke, "Literature Fingerprinting: A New Method for Visual Literary Analysis," 2007 IEEE Symposium on Visual Analytics Science and Technology, 2007, pp. 115-122.
